# Longevity of stainless steel crowns in molar incisor hypomineralization: a systematic review

**DOI:** 10.3389/fdmed.2026.1799014

**Published:** 2026-03-25

**Authors:** Nishi Joshi, Pratyasha Sharma, Srikala Bhandary, Manju Raman Nair

**Affiliations:** Department of Pediatric and Preventive Dentistry, NITTE (Deemed to be University) AB Shetty Memorial Institute of Dental Sciences, Mangaluru, India

**Keywords:** molar incisor hypomineralization, pediatric dentistry, permanent first molars, restorative outcomes, stainless steel crowns, systematic review

## Abstract

**Background:**

Molar incisor hypomineralization (MIH) is a developmental enamel defect frequently affecting permanent first molars, often leading to hypersensitivity, post-eruptive breakdown, and restorative failure. The compromised mechanical and adhesive properties of hypomineralized enamel make restorative management challenging, particularly in severe cases. Stainless steel crowns (SSCs) are widely advocated for full coronal coverage in such teeth; however, variability in reported outcomes and the increasing use of alternative restorative materials necessitate a systematic evaluation of their clinical performance.

**Objective:**

This systematic review aimed to compare the survival and clinical success of stainless steel crowns with alternative restorative approaches in the management of severely MIH-affected permanent molars in children and adolescents.

**Methods:**

The review was conducted in accordance with PRISMA guidelines and the Cochrane Handbook for Systematic Reviews of Interventions. Electronic searches were performed in PubMed, Scopus, Web of Science, and the Cochrane Library up to December 2025. Studies involving children aged 6–15 years with severe MIH affecting permanent first molars were included. Interventions comprising SSCs were compared with composite restorations and other full-coverage crowns. Eligible study designs included randomized controlled trials, retrospective studies, and clinical observational studies. Risk of bias was assessed using the Newcastle–Ottawa Scale. Due to heterogeneity among studies, a qualitative narrative synthesis was performed.

**Results:**

Five studies met the inclusion criteria. Across all included studies, stainless steel crowns demonstrated consistently higher survival and success rates compared with composite restorations and ceramic crowns over follow-up periods ranging from 12 to 24 months. SSCs showed superior retention, marginal integrity, and reduced need for retreatment, particularly in teeth with extensive enamel breakdown and hypersensitivity. Alternative restorations, especially multi-surface composite restorations, exhibited higher failure rates over time.

**Conclusion:**

Within the limitations of the available evidence, stainless steel crowns exhibit superior clinical survival and success compared with alternative restorative techniques in the management of severely MIH-affected permanent molars. SSCs remain a reliable, durable, and cost-effective treatment option for children with severe MIH. Further high-quality randomized controlled trials with longer follow-up periods and standardized outcome measures are required to strengthen the evidence base.

**Systematic Review Registration:**

PROSPERO CRD420251274395.

## Introduction

In 2001, Weerheijm and colleagues of the European Academy of Paediatric Dentistry (EAPD) introduced the term “Molar Incisor Hypomineralization” (MIH) to describe a systemic developmental enamel defect predominantly involving one to four first permanent molars, with possible involvement of the permanent incisors. The first comprehensive description of its clinical features was published in 2003 (1). MIH is associated with hypersensitivity, difficulty gaining adequate anaesthesia, atypical carious lesions, post-eruptive breakdown (PEB), a reduction in resin bond strength, aesthetic concerns and a reduction in quality of life. Although MIH primarily denotes hypomineralization affecting permanent first molars and incisors, similar defects have also been reported in the cusps of canines as well as in permanent second molars and premolars. When the incisors are not involved, the condition is referred to as molar hypomineralization (MH) (2, 3).

The etiology of the condition is considered multifactorial, involving the combined or additive influence of systemic, genetic, and epigenetic factors (4), and is closely linked to maternal and child health during the perinatal period. In this context, special emphasis should be placed on early-life oral microbiome development (5), as well as the concurrent presence of syndromes and genetic mutations that affect orofacial structures (6). In MIH-affected molars that are susceptible to post-eruptive breakdown (PEB) or hypersensitivity, early protective coverage is recommended to alleviate sensitivity, prevent secondary caries, and minimize the risk of PEB, which arises from the increased porosity and reduced mechanical properties of the affected enamel (7).

The management of hypomineralized dental enamel is primarily determined by the type and severity of the defect, which may be classified as mild, moderate, or severe. Among these, the severe form of MIH—characterized by the involvement of two or more cusps, with or without pulpal involvement—poses the greatest clinical challenge due to the limited range of effective treatment options available.

A major concern associated with the atypical restoration of severely affected hypomineralized teeth (SAHT) is the recurrent failure of cavosurface margins, often leading to repeated restoration replacement. Although adhesion between restorative materials and hypomineralized enamel (HE) may initially occur when clinically sound-appearing opaque enamel is preserved during cavity preparation, this can subsequently result in restoration disintegration. Furthermore, failure affecting even a single surface may necessitate replacement of the entire restoration. Consequently, SAHT frequently require full-coverage crowns, which have demonstrated superior clinical success rates compared to multi-surface restorations (8, 9).

A wide range of treatment options are available for teeth affected by MIH, involving both molars and incisors. These approaches include preventive measures, restorative interventions, and, in severe cases, extraction followed by possible orthodontic management. However, selecting the most appropriate treatment strategy remains challenging. Key factors influencing decision-making include the child's level of cooperation, the stage of dental development, and the severity of the enamel defect. In addition, patient and parental preferences, the presence of associated dental anomalies, and the potential psychosocial impact on the child should also be carefully considered.

Various restorative materials, including glass ionomer cements, composite resin (CR), compomers, stainless steel crowns (SSCs), and ceramics, have been proposed for the management of first permanent molars severely affected by MIH.

However, current scientific evidence does not clearly identify an optimal restorative material of choice. Among the available options, stainless steel crowns (SSCs) are regarded as cost-effective, durable, and associated with minimal technique sensitivity, making them a commonly recommended treatment modality for severely hypomineralized molars (10). Despite their widespread clinical use, variability in reported outcomes and the emergence of alternative restorative techniques necessitate a comprehensive evaluation of their clinical performance. Therefore, this systematic review aims to compare the success rates of stainless steel crowns with those of other restorative approaches used in the management of hypomineralized molars.

## Methodology

The systematic review was conducted in accordance with the PRISMA (Preferred Reporting Items for Systematic Reviews and Meta-Analyses) statement (11) and the guidelines outlined in the Cochrane Handbook for Systematic Reviews of Interventions (12). A PICO (Population, Intervention, Comparison, and Outcome) framework was employed to formulate a focused research question and to define the inclusion and exclusion criteria for the present study (13). Registration in the PROSPERO database was completed following the screening phase (CRD420251274395).

### Inclusion and exclusion criteria for study selection

The inclusion criteria for selecting studies in the PICOS frame work was:

Population (P): studies involving children aged 6–15 years with MIH teeth were considered.
Intervention (I): studies that employed minimal and full coverage restorations for severe cases of MIH were considered.
Comparison (C): studies that compared Stainless steel crowns with composite restorations or other crowns such as zirconia, cast metal etc were considered.
Outcome(O): to assess success rate of stainless steel crowns over other restorations and crowns in severe cases of Hypomineralized molars.
Study Design (S): randomized controlled trials, cross-sectional studies, retrospective studies, clinical studies.

### Literature search

Multiple electronic databases, including PubMed, Web of Science, Scopus, Cochrane Library were searched using a combination of keywords and Medical Subject Headings (MeSH) terms until, December 2025 (Table 1).

**Table 1:** Search strategy and search terms used.

Database	Search query	Results
PubMed	((child OR children OR pediatric OR paediatric OR adolescent) AND (permanent first molar OR molar incisor hypomineralization OR MIH) AND (stainless steel crown OR SSC) AND (composite restoration OR zirconia crown OR ceramic crown OR full coverage restoration) AND (survival OR success OR longevity OR clinical outcome OR follow-up))	25
Scopus	(child OR pediatric OR pediatric OR adolescent ANDMIH OR “molar incisor hypomineralization” OR “permanent first molar” AND “stainless steel crown” OR SSC AND composite OR zirconia OR ceramic OR “full coverage” AND survival OR success OR longevity OR follow-up)	5
Web of science	((child OR pediatric OR paediatric OR adolescent) AND (“molar incisor hypomineralization” OR MIH OR “permanent first molar”) AND (“stainless steel crown” OR SSC) AND (composite restoration OR zirconia crown OR ceramic crown OR “full coverage restoration”) AND (survival OR success OR longevity OR “clinical outcome” OR follow-up))	10
Cochrane library	TITLE-ABS-KEY(child OR children OR pediatric OR paediatric OR adolescent) AND (permanent first molar OR molar incisor hypomineralization OR MIH) AND (stainless steel crown OR SSC) AND (composite restoration OR zirconia crown OR ceramic crown OR full coverage restoration) AND (survival OR success OR longevity OR clinical outcome OR follow-up)	16

The search strategy combined terms related to Molar Incisor Hypomineralization, Permanent first molars, stainless steel crowns, zirconia crowns, composite restorations, success rates. Boolean operators (AND, OR) were used to combine the search terms effectively. No filters or date restrictions were placed with only English language publications considered for inclusion.

The initial search retrieved 56 potential articles. After the duplicates were removed, 24 articles remained; the titles and abstracts of these 24 articles were read, resulting in the selection of 32 articles for reading the full text. The data of the 5 included articles were extracted.

### Eligibility criteria

#### Inclusion criteria

- Population: Children and adolescents aged 6–15 years
- Intervention: Full coverage restorations for severe MIH cases
- Comparison: SSCs with composite or other crowns.
- Outcomes: success rate of stainless steel crowns over other restorations.
- Study designs: randomized controlled trials, cross-sectional studies, retrospective studies, clinical studies.
- Language: English language articles only.

#### Exclusion criteria

- Case reports or case series
- Reviews, editorials, conference abstracts, and expert opinions
- Animal or *in vitro* studies
- Studies without clearly reported outcomes
- Studies involving adults only

### Data extraction

The following information was collected and tabulated:

- Author and year
- Sample size
- Age group and gender
- Severity of MIH
- Type of restoration done
- Follow up period

Two reviewers (N.J. and P.S.) independently screened titles and abstracts for eligibility. Full texts of potentially eligible articles were assessed independently. Disagreements were resolved through discussion, and where necessary, consultation with a third and fourth reviewers (S.B. and M.R.N.). The detailed characteristics of each of the included studies is explained in Table 2. Due to the heterogeneity of the studies and lack of quantitative outcomes in the included studies, a narrative synthesis was done. Possible sources of heterogeneity among included studies were explored qualitatively through structured subgroup comparisons. Studies were grouped and compared based on study design, age group of participants, and type of intervention done for severe MIH cases. Differences in comparator technique were examined across these groups to identify consistent patterns and potential sources of variability. Meta-regression was not performed due to the absence of pooled quantitative data.

**Table 2:** Characteristics of included articles.

Sr no. 1	Author, year	Sample size	Age group (years)	Gender	Severity of MIH	Type of restorative intervention	Follow up period
1.	Zagdwon et al. 2003 (18)	17 patients, 42 restorations	6–13 years	11 female, 7 male	Severe	SSCs and cast adhesive coping	24 months
2.	Talekar et al. 2023 (19)	72 teeth	6–12 years	Not described	Severe	SSCs and zirconia	12 months
3.	Farias et al. 2021 (10)	115 teeth	7–10 years	51% male 49% female	Severe	SSCs and composite	24 months
4.	Singh et al. 2021 (20)	46 children, 60 teeth	8–15 years	Not described	Severe	SSCs, zirconia and emax	24 months
5.	Geduk et al. 2023 (21)	20 children, 48 teeth	6–13 years	Not described	Severe	SSCs and Zirconia	18 months

### Risk of bias assessment

**Figure 1:**
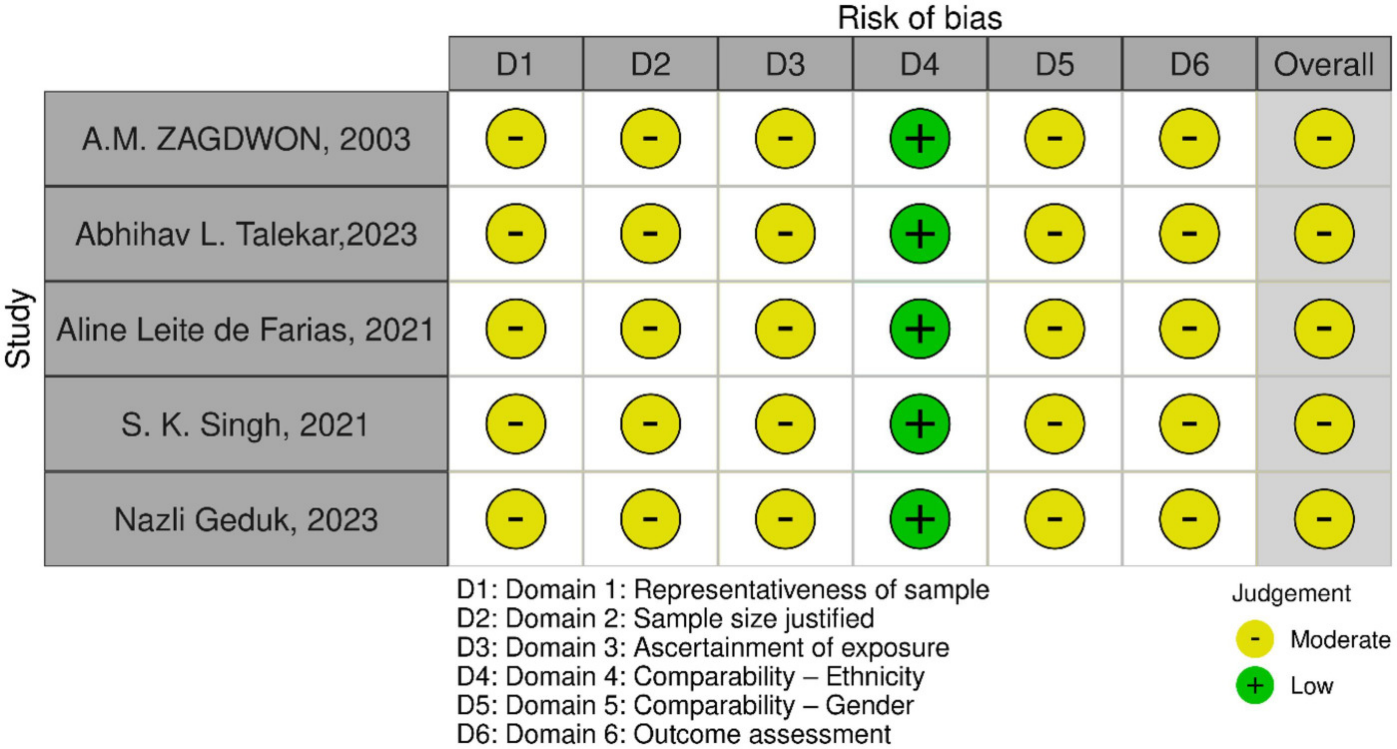
Quality of included studies assessed with Newcastle-Ottawa scale.

The quality of included studies was independently assessed by two reviewers (N.J. and P.S) with the Newcastle-Ottawa Scale (14) and the Robvis tool (15) was used to generate traffic light plots for the same (Figure 1) any discrepancies were resolved by consensus and cross-checked by a third and fourth reviewers (S.B. and M.R.N.).

## Results

**Figure 2:**
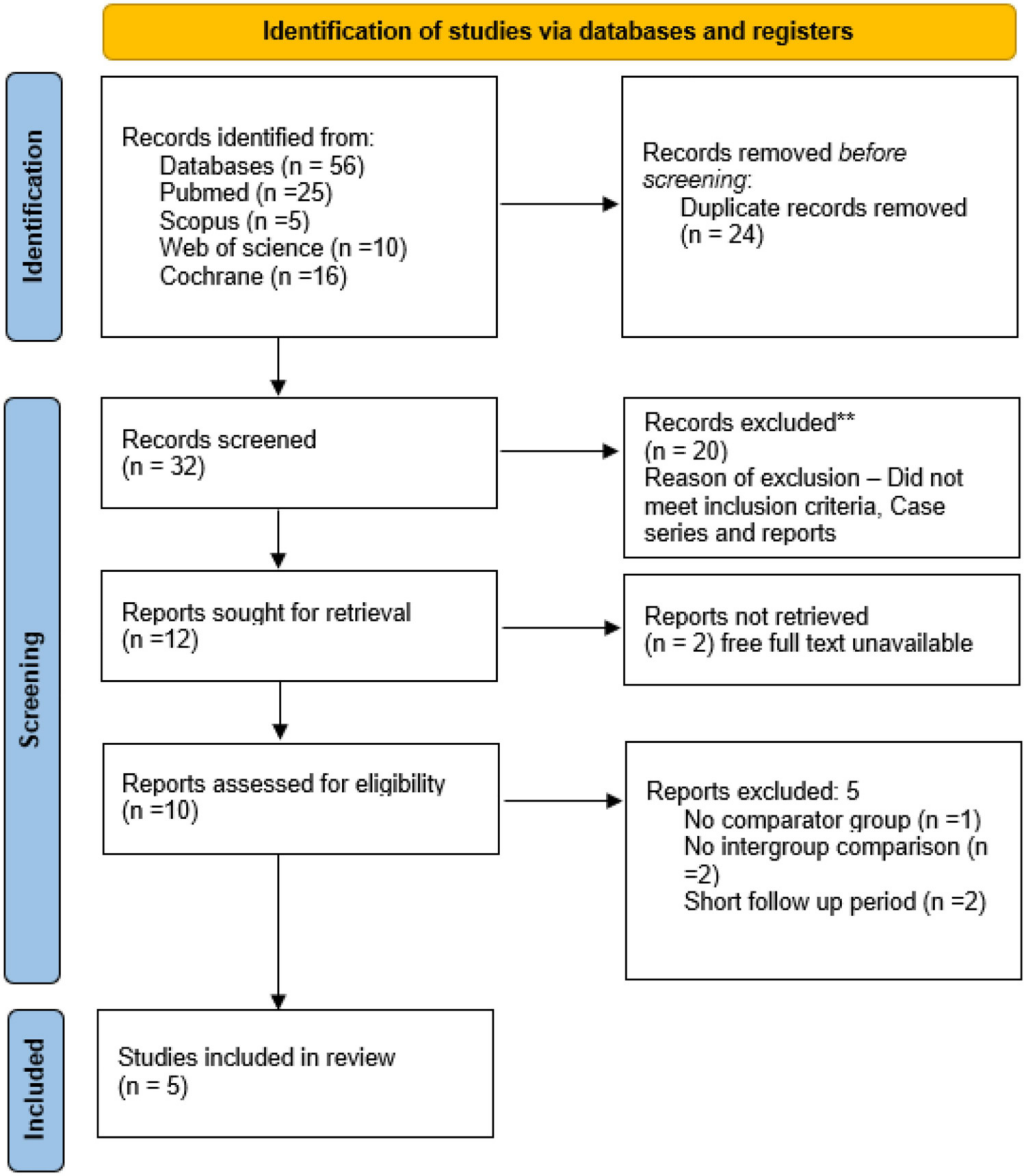
PRISMA flow diagram.

The PRISMA flow diagram illustrating the study selection process is presented in Figure 2 (16). The initial electronic search across four databases yielded a total of 56 records. Following the removal of duplicates, 24 articles remained and were screened based on titles and abstracts. Of these, 32 studies were considered potentially relevant and underwent full-text assessment. After detailed evaluation, five studies met the predefined inclusion criteria and were included in the final qualitative synthesis.

Several studies initially appeared to be eligible but were excluded after full-text review for not fulfilling the inclusion criteria. Studies were excluded if they were narrative reviews, case reports, case series, or *in vitro* studies, as these did not provide original clinical outcome data. Additional exclusions included studies involving adult populations only, those lacking clearly defined restorative outcomes, and studies that did not specifically evaluate full-coverage restorations or compare stainless steel crowns with alternative restorative techniques in MIH-affected permanent molars.

The characteristics of the included studies are summarized in Table 2. The included studies comprised randomized controlled trials, retrospective studies, and clinical observational studies, with participants ranging in age from 6 to 15 years. All studies evaluated teeth affected by severe MIH, primarily involving permanent first molars, and compared stainless steel crowns with alternative restorative materials such as composite resin and zirconia crowns. Follow-up periods varied across studies, ranging from 12 to 24 months.

Qualitative subgroup comparisons revealed several sources of heterogeneity among the included studies. Differences were observed in study design, sample size, follow-up duration, and comparator restorative materials. Studies conducted in clinical settings generally reported higher survival and success rates for stainless steel crowns compared with multi-surface restorations, particularly composite resin restorations, which demonstrated higher failure rates over time. (Table 3) Age-related differences were minimal, as comparable clinical outcomes were reported across school-aged children and adolescents.

**Table 3:** Survival and clinical outcomes of included studies.

Study (Author, Year)	Follow-up	SSC survival rate	Comparator & survival rate	Number of failures (SSC vs. Comparator)	Statistical significance
Zagdwon et al. 2003 (18)	24 months	100%	Cast adhesive coping – Not explicitly separated	0 SSC failures reported	Not clearly reported
Talekar et al. 2023 (19)	12 months	Higher than zirconia	Zirconia – lower survival than SSC	Fewer failures in SSC group	Statistically significant
Farias et al, 2021 (10)	24 months	**94.4%**	Composite resin – **49.2%**	SSC: 3 failures (5.6%) CR: 31 failures (50.8%)	Statistically significant
Singh et al. 2021 (20)	24 months	Full cast metal showed high retention	Zirconia & Lithium disilicate – similar clinical success	Failures not significantly different	No statistically significant difference
Geduk et al. 2023 (21)	18 months	**100%**	Zirconia – **95.2%**	SSC: 0 failures Zirconia: 1 failure	No statistically significant difference

Despite methodological and clinical variability, a consistent pattern emerged across all included studies, indicating superior survival and clinical success of stainless steel crowns when compared with other restorative techniques in severely hypomineralized molars. These findings were particularly evident in teeth with extensive enamel breakdown or hypersensitivity, where full-coverage restorations demonstrated improved durability and reduced need for retreatment.

## Discussion

This systematic review aimed to evaluate and compare the survival and clinical success of stainless steel crowns (SSCs) with alternative restorative approaches in the management of severely hypomineralized permanent molars. The findings of this review indicate that SSCs consistently demonstrate superior survival rates and clinical performance when compared with other restorative modalities, including composite resin and ceramic crowns, in children affected by severe MIH.

The restorative management of MIH-affected molars remains challenging due to the compromised structural integrity of hypomineralized enamel, which is characterized by increased porosity, reduced mineral content, and inferior mechanical properties (1, 17). These characteristics predispose affected teeth to post-eruptive breakdown, hypersensitivity, and restoration failure, particularly when adhesive restorative materials are used. The difficulty in achieving reliable adhesion to hypomineralized enamel has been well documented and contributes to the frequent marginal breakdown and loss of restorations reported in multi-surface composite restorations (8, 9).

Beyond material properties, several clinical and procedural factors may significantly influence the survival and success of SSCs in MIH-affected permanent molars. The clinical technique employed regarding conventional crown preparation or alternative approaches such as the Hall technique, may affect outcomes. Conventional preparation typically involves occlusal reduction and circumferential tooth preparation to ensure adequate seating and marginal adaptation, whereas modified or minimally invasive approaches aim to preserve tooth structure and reduce patient discomfort. In severely hypomineralized molars with extensive enamel breakdown, adequate occlusal reduction and removal of unsupported enamel are critical to ensure proper crown fit and prevent occlusal discrepancies.

Crown adaptation procedures, including contouring and crimping, are equally important determinants of clinical longevity. Proper marginal adaptation enhances retention, reduces microleakage, and minimizes cement washout, thereby contributing to improved survival rates. Inadequate adaptation may compromise marginal integrity and predispose to plaque accumulation or gingival irritation. The relatively forgiving nature of SSCs in terms of technique sensitivity may partly explain their consistent performance across the included studies (18–21).

The type of luting cement and the quality of isolation during cementation also play a crucial role in clinical outcomes. Given the hypersensitivity and difficulty in achieving profound anesthesia in MIH-affected molars, maintaining adequate moisture control can be challenging (17). Glass ionomer cements are commonly used due to their fluoride release and chemical adhesion; however, improper isolation may affect cement integrity and long-term retention. Effective isolation protocols and careful cementation technique are therefore essential to optimize SSC survival.

Management of hypersensitivity and anesthesia is another relevant clinical consideration. MIH-affected molars frequently present with increased sensitivity and difficulty achieving adequate local anesthesia, which can complicate operative procedures.8 Proper pain control, behavioral management, and minimally traumatic tooth preparation contribute to improved patient cooperation and procedural success. In this context, SSCs may offer an advantage by providing rapid full coronal coverage, thereby reducing hypersensitivity and protecting the tooth from further structural breakdown.

Among the included studies, Zagdwon et al. reported favorable outcomes with SSCs and cast adhesive copings in severely affected molars over a 24-month follow-up period, highlighting the durability of full-coverage restorations in managing extensive enamel defects (18). Similarly, the clinical studies by Talekar et al. and Geduk et al. demonstrated higher survival rates for SSCs compared with zirconia crowns, particularly in younger children where moisture control and patient cooperation may be limited (19, 21). These findings support earlier clinical recommendations that advocate SSCs as a predictable and pragmatic treatment option for severely affected molars in pediatric populations (9, 17)..

The study by de Farias et al. provided comparative evidence indicating significantly higher survival rates for SSCs than composite resin restorations in MIH-affected molars over a 24-month period. Composite restorations, while esthetically acceptable, were associated with higher failure rates due to marginal degradation, secondary caries, and restoration loss. This reinforces the notion that adhesive restorations may be less suitable for severely hypomineralized enamel, particularly in load-bearing posterior teeth (10).

Similarly, Singh et al. evaluated SSCs, zirconia crowns, and lithium disilicate (Emax) restorations and reported greater clinical reliability with SSCs in terms of retention and marginal integrity. Although ceramic crowns offer improved esthetics, their technique sensitivity, higher cost, and need for greater tooth reduction may limit their applicability in young patients with severe MIH. In contrast, SSCs provide full coronal coverage with minimal tooth preparation, reducing the risk of further enamel loss and pulp involvement (20).

Despite variations in study design, follow-up duration, and comparator materials, the findings across all included studies were remarkably consistent, favoring SSCs as the most reliable restorative option for severe MIH. These results align with existing literature emphasizing the role of SSCs in reducing hypersensitivity, preventing post-eruptive breakdown, and minimizing the need for repeated restorative interventions (9, 17).

Nevertheless, the strengths of this review include adherence to PRISMA guidelines (11), comprehensive database searching, and rigorous risk of bias assessment using the Newcastle–Ottawa Scale. By focusing specifically on severe MIH cases and full-coverage restorations, this review provides clinically relevant guidance for pediatric dentists managing this challenging condition.

## Conclusion

Within the limitations of the available evidence, stainless steel crowns demonstrate superior survival and clinical success compared with alternative restorative techniques in the management of severely hypomineralized permanent molars. Their durability, cost-effectiveness, and reduced technique sensitivity make SSCs a reliable first-line treatment option for children with severe MIH. Future well-designed randomized controlled trials with longer follow-up periods and standardized outcome measures are required to further validate these findings and explore patient-centered outcomes.

## Limitations

This systematic review included only five studies, which reflects the currently limited body of clinical evidence specifically evaluating stainless steel crowns in severely MIH-affected permanent molars. The limited number of eligible studies diminishes the overall strength and certainty of the available evidence and consequently affects the robustness of the conclusions. Substantial heterogeneity across studies, regarding study design, sample size, duration of follow-up, comparator restorative materials, and outcome assessment, prevented quantitative meta-analysis and the derivation of pooled effect estimates. Moreover, the predominantly short- to medium-term follow-up periods (12–24 months) limit the ability to extrapolate findings to long-term clinical performance. Collectively, these limitations may influence both the generalizability and interpretative strength of the conclusions. Although the current evidence indicates favorable outcomes for stainless steel crowns in severe MIH, further high-quality, adequately powered randomized controlled trials with standardized outcome measures and extended follow-up are essential to reinforce the evidence base.

## Data Availability

The datasets presented in this study can be found in online repositories. The names of the repository/repositories and accession number(s) can be found below: https://www.crd.york.ac.uk/PROSPERO/view/CRD420251274395.

## Clinical implication and future scope

The evidence synthesized in this review reinforces the role of stainless steel crowns (SSCs) as a dependable and clinically predictable restorative option for severely MIH-affected permanent molars, particularly in pediatric populations where restoration longevity and reduced technique sensitivity are paramount. SSCs may therefore be considered a primary treatment modality for teeth presenting with extensive post-eruptive enamel breakdown or pronounced hypersensitivity, given their capacity to minimize restoration failure and the need for retreatment. Future investigations should prioritize adequately powered, multicenter randomized controlled trials with prolonged follow-up and standardized outcome criteria. In addition, research should systematically evaluate specific clinical and procedural determinants of SSC longevity, including preparation design, degree of occlusal and proximal reduction, crown adaptation techniques, choice of luting cement, isolation methods, and perioperative management of hypersensitivity and anesthesia, to refine clinical protocols and enhance long-term treatment outcomes.
